# Advances in viticulture *via* smart phenotyping: current progress and future directions in tackling soil copper accumulation

**DOI:** 10.3389/fpls.2024.1459670

**Published:** 2024-11-04

**Authors:** Youry Pii, Guido Orzes, Fabrizio Mazzetto, Paolo Sambo, Stefano Cesco

**Affiliations:** ^1^ Faculty of Agricultural, Environmental and Food Sciences, Free University of Bolzano, Bolzano, Italy; ^2^ Faculty of Engineering, Free University of Bolzano, Bolzano, Italy; ^3^ Competence Center for Plant Health, Free University of Bolzano, Bolzano, Italy; ^4^ Department of Agronomy, Food, Natural Resources, Animals and Environment (DAFNAE), University of Padova, Legnaro, Italy

**Keywords:** viticulture, Cu toxicity, smart phenotyping, data fusion, artificial intelligence

## Abstract

Modern viticulture faces significant challenges including climate change and increasing crop diseases, necessitating sustainable solutions to reduce fungicide use and mitigate soil health risks, particularly from copper accumulation. Advances in plant phenomics are essential for evaluating and tracking phenotypic traits under environmental stress, aiding in selecting resilient vine varieties. However, current methods are limited, hindering effective integration with genomic data for breeding purposes. Remote sensing technologies provide efficient, non-destructive methods for measuring biophysical and biochemical traits of plants, offering detailed insights into their physiological and nutritional state, surpassing traditional methods. Smart phenotyping is essential for selecting crop varieties with desired traits, such as pathogen-resilient vine varieties, tolerant to altered soil fertility including copper toxicity. Identifying plants with typical copper toxicity symptoms under high soil copper levels is straightforward, but it becomes complex with supra-optimal, already toxic, copper levels common in vineyard soils. This can induce multiple stress responses and interferes with nutrient acquisition, leading to ambiguous visual symptoms. Characterizing resilience to copper toxicity in vine plants *via* smart phenotyping is feasible by relating smart data with physiological assessments, supported by trained professionals who can identify primary stressors. However, complexities increase with more data sources and uncertainties in symptom interpretations. This suggests that artificial intelligence could be valuable in enhancing decision support in viticulture. While smart technologies, powered by artificial intelligence, provide significant benefits in evaluating traits and response times, the uncertainties in interpreting complex symptoms (e.g., copper toxicity) still highlight the need for human oversight in making final decisions.

## General aspects and introduction to the main viticulture challenges

Modern agricultural practices face the urgent challenge of significantly increasing crop yield to meet the growing global food demand of a population projected to reach 9 billion people by 2050 (FAO, 2009; Tilman et al., 2011). However, phenomena like soil degradation (*e.g.*, erosion, salinization, and pollution) and urbanization are diminishing the total agricultural land area, potentially leading to reduced productivity (Ferreira et al., 2022), posing also a threat for vital soil ecosystem services (AbdelRahman, 2023; Rathore et al., 2023). This scenario is further exacerbated by climate change and global warming, leading to increasing abiotic and biotic stress on crop plants (Raza et al., 2019), a trend clearly observed in viticulture in recent decades. Notably, recent years have been characterized by a significant resurgence in pathogenic infections of grapevines (Bardsley et al., 2023), such as downy mildew (*Plasmopara viticola*) and powdery mildew (*Erysiphe necator*) (Rienth et al., 2021). To address this issue, the use of fungicides has been increasingly relied upon to protect crops and secure yields (Wong et al., 2001; Gessler et al., 2011; Fontaine et al., 2019). However, beside its lack of long-term sustainability, this approach has led to a gradual accumulation of fungicidal residues in vineyard soils, posing a serious threat to soil health, as described for the case of copper (Cu) (Brunetto et al., 2016). In fact, the soil concentration of this metal, which naturally ranges from 2 to 50 mg kg^-1^ (Oorts, 2013), can occasionally exceed 1000 mg kg^−1^ due to agricultural inputs including Cu-based fungicides, notably observed in vineyard soils in France and Brazil (Brunetto et al., 2016). This phenomenon not only poses a serious threat to environmental integrity but also hinders vineyard replanting. Severe instances of Cu toxicity in grafted cuttings intended for vineyard rejuvenation have become increasingly common in certain viticulture regions (Cesco et al., 2021). This represents a significant challenge, particularly considering the substantial contribution of this production sector to the overall livelihood of the agricultural context (Montalvo-Falcón et al., 2023). Hence, there is a pressing need to implement targeted measures to enhance vine resilience against both pathogens attacks and alterations in soil fertility. For pathogen resistance, various initiatives—ranging from traditional breeding methods to advanced assisted evolution techniques and biotechnological strategies—have been successfully implemented or are currently underway (Cesco et al., 2021). However, efforts to develop vine plant materials specifically adapted to soils with compromised fertility, such as those with Cu accumulation, remain relatively limited. Furthermore, a comprehensive evaluation of the performance of pathogen-resistant vine varieties under altered soil fertility conditions is still needed.

## Cu in viticultural soils and its toxic effects in vine plants

Although Cu is an essential nutrient, when present in excess in the soil, vine plants exhibit various phenotypic responses as adaptive mechanisms to cope with toxicity and ensure growth and survival (Nagajyoti et al., 2010; Cesco et al., 2021). These responses, whose intensity and severity progressively increase as the levels of Cu available fractions in soil increase, include alterations in morphology (*e.g.*, limited root development, stunted shoot growth, and leaf chlorosis), physiology (*e.g.*, activation of antioxidant defense systems, accumulation of osmoprotectants, enhanced chelation of metals *via* phytochelatins and metallothioneins), and biochemistry (enhanced synthesis of metal-binding proteins and detoxification enzymes) (Cesco et al., 2021, 2022; Kosakivska et al., 2021). Furthermore, when pronounced, soil Cu accumulation impairs chlorophyll synthesis pathways, resulting in decreased photosynthetic efficiency and chlorotic canopy appearance. Symptomatically, in grapevine tissues Cu accumulation can lead to necrosis, wilting, and stunted growth, impairing overall plant vigor and productivity (Brunetto et al., 2016). Eventually, Cu toxicity may alter fruit development and quality, leading to decreased yield and compromised wine characteristics (Cambrollé et al., 2013, 2015). In this regard, it is important to note that these severe symptoms are typically observed in plants experiencing severe Cu-toxicity conditions in soils (Cesco et al., 2021). Conversely, moderate or latent ones often induce the manifestation of milder symptoms that may not always be readily and unequivocally identifiable.

Interestingly, several pieces of research have highlighted that, among its various effects, Cu toxicity can also antagonize the acquisition of other essential micro and macronutrients, in different plant species, as for instance Arabidopsis (Hippler et al., 2018), alfalfa, lettuce (Hong et al., 2015), sorghum (Roy et al., 2017), grapevine (Toselli et al., 2009; Baldi et al., 2018), Citrus (Hippler et al., 2016) and poplar (Tőzsér et al., 2023). Recent evidence has suggested that this competition might be ascribable to an impairment in the functionality of root mechanisms involved in acquiring essential nutrients. In particular, despite the antagonism between Cu and phosphorus (P) being known in poplar and grapevine for a while (Teng and Timmer, 1990; Toselli et al., 2009; Baldi et al., 2018), high concentrations of Cu were just recently demonstrated to affect the biochemical mechanisms underlying P acquisition (Feil et al., 2020). Additionally, Cu may interfere with cucumber plants ability to induce the molecular machineries involved in nitrate uptake in the high affinity range (Feil et al., 2023), impairing the generation of the transmembrane proton gradient required for the nitrate uptake mechanism. Furthermore, a detailed investigation on grapevine rootstocks exposed to Cu toxicity highlighted that the differential responses were mainly ascribable to fine tuning of bivalent cations uptake and allocation mechanisms (Marastoni et al., 2019b). Interestingly, manganese (Mn) deficiency induced by Cu toxicity has been described in the most sensitive rootstock, demonstrating an antagonism between the two elements (Marastoni et al., 2019b). This aspect is particularly critical in pathogen-resilient vine cultivars. Despite expressing resistance genes, these cultivars still rely on a pathogen response mechanism that involves local increases in the concentration of several nutrients, Mn among them, which are crucial for synthesizing secondary metabolites and reactive oxygen species (Cesco et al., 2020). Taken together, these recent findings indicate that Cu excess in soil not only induces typical leaf symptoms at different degrees of severity but also significantly disrupts the functionality of nutrient acquisition mechanisms in roots. This disruption can then lead to deficiency situations, even if at latent levels.

## Plant phenomics and phenotyping

Plants, as sessile organisms, must adapt their morphological and physiological characteristics to cope with the environmental changes (Foyer and Kranner, 2023). In this respect, crop plants will be significantly impacted by the worsening climate conditions and altered soil fertility, influencing the genotype-environment interaction and affecting the expression of plant phenotypes (Gupta et al., 2015). Therefore, to evaluate the potential of different genotypes, it is essential to monitor changes in plants phenotype under environmental stressors, either in field or controlled conditions, to finally unravel the regulatory networks involved in stress responses (Mickelbart et al., 2015). This aspect becomes more relevant in the post-genomic era, where the genomic resources should assist the selection of the most suitable and potentially successful crop varieties. However, despite advancements of next-generation sequencing and genotyping, the lack of accurate phenotype data acquisition hinders the utilization of genomic knowledge for breeding purposes (Furbank et al., 2019; Yang et al., 2020). In this respect, it should be highlighted that *plants phenomics* is defined as the high-throughput and accurate acquisition of data regarding plant phenotypes (Houle et al., 2010), while phenotyping refers to a set of methods and protocols aimed at monitoring plant traits under varying environmental conditions, with specified accuracy and precision at different scales, ranging from single organ to whole canopy (Fiorani and Schurr, 2013; Mahlein, 2016). Until recently, methods for assessing plant phenotypes have relied on destructive, labor-intensive and time-consuming techniques (Zhao et al., 2019; Yang et al., 2020; Singh et al., 2021). Therefore, there is a pressing need to develop non-destructive methods that can accurately, objectively, and efficiently assess plants’ phenotypic traits in response to abiotic and/or biotic stresses (Zhao et al., 2019; Yang et al., 2020; Singh et al., 2021). In this context, high-throughput phenotyping could play a crucial role in facilitating breeders’ efforts to develop novel and resilient genotypes across multiple environments (Crossa et al., 2017). However, the advancement of phenomics currently lags behind genomics, which poses a limitation in applying these approaches to assess crop genotype performance (Bohra et al., 2021). Concerning grapevine and viticulture, current progress in smart phenotyping includes advancements in automated data collection and analysis, enabling more accurate assessments of plant health, growth patterns, and environmental responses. In this respect, it is noteworthy the application of drones equipped with multispectral imaging monitor vineyards, detecting plant health issues like water stress, pests, and nutrient deficiencies (Campos et al., 2021). In addition, robots and sensors have also been exploited to measure vineyard canopy traits, aiding pruning decisions, crop load management, and improving vine balance through automated data collection (Fernández-Novales et al., 2021). In the future, these technologies could contribute to precision viticulture by optimizing disease management, improving yield predictions, and reducing resource use, all of which will enhance the sustainability and efficiency of modern viticulture (Poblete-Echeverría and Tardaguila, 2022).

## Image-based technologies for plants’ phenotyping

The advancement of remote sensing technologies, such as Light Detection and Ranging (LiDAR) and hyperspectral/multispectral sensors, has significantly enhanced the development of rapid, non-destructive methods for measuring the biophysical and biochemical traits of plants. These advancements have made the identification and evaluation of crop variety performance much more efficient compared to traditional methods. These traits, which include indicators of plant physiological state, photosynthetic capacity, and nutrient stresses (Ali and Imran, 2021), were traditionally determined through field surveys, soil or leaf nutrient sampling, and climatology recording (Fournier and Hall, 2017). Image-based technologies have shown potential for phenotyping by enabling the real-time identification of alterations in plant reflectance, biomass, and thermal radiation at a high resolution, providing valuable information about their morphophysiological traits, growth and development. Pioneering experiences utilizing imaging techniques based on Red, Green and Blue (RGB) sensors as well as chlorophyll fluorescence (ChF) imaging sensors, have already been documented in the literature (Al-Tamimi et al., 2022).

From a functional perspective, remote sensing of vegetation traits works by capturing electromagnetic radiation that interacts with leaf or plant canopies, producing a spectral response curve. This response, which combines reflected, absorbed and transmitted or emitted radiation, is influenced by various factors. Pigments such as chlorophyll, carotenoids, and anthocyanins affect reflectance in the visible (VS) band (400–700 nm) and are, thus, detectable in this region (Merzlyak et al., 2003). In contrast, the ratio of mesophyll cells to intercellular air spaces per unit leaf area influences the near-infrared (NIR, 700–1000 nm) reflectance, allowing for the estimation of canopy structural parameters (Kattenborn et al., 2019). The thermal, or long-wave, infrared band (750–1400 nm) enables the measurement of canopy surface temperatures, canopy transpiration rates, and leaf or stomatal conductance (Ishimwe et al., 2014). Non-pigment biochemical contents, including water, nitrogen (N), protein, lignin, and cellulose, predominantly influence spectral reflectance into the micro or short-wave infrared (SWIR) range (1400–2500 nm) (Féret et al., 2019). One widely used spectral vegetation index is the Normalized Difference Vegetation Index (NDVI) (Jones and Vaughan, 2010; Calcante et al., 2012; Xue and Su, 2017), which is calculated as the normalized ratio between red and near-infrared bands from multispectral information. In more detail, the formula is provided below. It involves spectral radiance (or reflectance) measurements recorded by sensors in the red (visible) and NIR (near-infrared) regions (Huang et al., 2021).


$$\text{NDVI}=\frac{\text{NIR}-\text{red}}{\text{NIR}+\text{red}}$$

NDVI values range from -1 to +1 due to a normalization procedure, with different values corresponding to differences in spectral responses of objects/materials (such as water, wet soil, dry soil, and leaves) (Huang et al., 2021). Moreover, NDVI values have been linked to canopy structure, photosynthesis rates, and plant health (Gamon et al., 1995). However, a specific NDVI value does not have a singular interpretation (explicit meaning), as it is an index created to simplify complex relationships (Xue and Su, 2017). In addition to NDVI, numerous other vegetation indices combining visible light radiation and non-visible spectra have been proposed to obtain proxy quantifications of the vegetation cover, vigor, and growth dynamics (see Xue and Su, 2017 for a detailed review). Among such indices, it is worth mentioning the Plant Senescence Reflectance Index (PSRI - Merzlyak et al., 2003), which assesses the carotenoids to chlorophyll ratio, the Green Leaf Index (GLI - Louhaichi et al., 2001), which is particularly suitable to determine leaf chlorophyll, and the Green-Red Vegetation Index (GRVI - Tucker, 1979), a useful indicator of vegetation phenology, especially for leaf autumn coloring, but also disturbance and ecosystem types.


$$PSRI=\frac{red-green}{NIR}$$

$$GLI=\frac{2\;green-red-blue}{2\;green+red+blue}$$

$$GRVI=\frac{green-red}{green+red}$$

Since vegetation indices have different effectiveness in monitoring the spatiotemporal content of chlorophyll, carotenoids and nitrogen, the integration of several detection methods and indices related to the different bands of the leaf spectral signature is advisable (Nestola et al., 2018).

Remote sensing can be conducted using various supports, including spaceborne (micro- and nano-) satellites, airborne platforms, drones, and ground-based (hand-held) devices. Examples of critical analysis of these applications with crops are the experience to monitor i) fruit-crop diseases in orchards (Zhang et al., 2021), ii) fruit and canopy traits of *Citrus sinensis* orchards (Ali and Imran, 2021) and iii) leaf nitrogen content in apple trees (Zhang et al., 2012).

Experiences of yield predictions by using remote sensing techniques (He et al., 2022) and orchard trees identification through imaging techniques and vegetation indices (Ozdarici-ok and Ok, 2023) have been also reported. Furthermore, such indices can also be effectively adopted to track photosynthetic phenology, which allows in turn the modeling of CO_2_ fluxes as well as forest conditions and functionality (Nestola et al., 2018; Guo et al., 2024). Remote sensing technologies are increasingly being applied in viticulture to improve vineyard management by providing valuable data for decision-making. These technologies use satellite imagery, drones, and sensors to monitor various aspects of the vineyard without direct contact, offering real-time information that can optimize viticulture practices. Specific experiences in which remote sensing technologies have been applied to vineyards and viticulture are reported in **Table 1**.

**Table 1:** Specific examples of remote sensing technologies applied to viticulture.

Remote Sensing Technology	Application in viticulture	Reference
Normalized Difference Vegetation Index (NDVI)	Grapevine canopy mapping	(De Castro et al., 2018)
	Vigour maps and long-term monitoring	(Nonni et al., 2018)
	Monitoring of canopy health and vigour	(Mazzetto et al., 2010)
	Monitoring vine water status, yield, and berry composition	(Kotsaki et al., 2019)
	Yield estimation	(Hacking et al., 2019)
	Characterization of vine foliage	(Milella and Reina, 2024)
	Diagnosis of *Plasmopara viticola* in vine	(Calcante et al., 2011)
	Total Leaf Area assessment	(Vélez et al., 2020)
⁠Multispectral and Hyperspectral Imaging	Assessment of vegetative, productive, and berry composition spatial variability within a vineyard	(Rey-Caramés et al., 2015)
	Assessment of biophysical and geometrical parameters at grapevine scale	(Pádua et al., 2020)
	Assessment of geometrical parameters at grapevine scale	(Sousa et al., 2022)
	Assessment of water stress index	(Buunk et al., 2023)
	Investigation of grapevine photosynthetic parameters	(Ozelkan et al., 2015)
	Prediction of degree brix in wine grapes	(Swe et al., 2023)
	*In-situ* optical monitoring of grape ripening	(Oliveira et al., 2024)
	Determination of biophysical variables	(Darra et al., 2021)
Thermal Imaging	Prediction of grapevine canopy nitrogen status	(Walker et al., 2021)
	Monitoring of water use and stress in vineyards	(Knipper et al., 2019)
	Detection of biotic or abiotic stress in vineyards	(Fevgas et al., 2023)
⁠Light Detection and Ranging (LiDAR)	Canopy size assessment	(Pagliai et al., 2022)
	Automatic detection of vine rows	(Biglia et al., 2022)
	Automatic grapevine trunk detection	(Jurado et al., 2020)
Satellite Imagery	Assessment of vineyards soil erosion	(Straffelini et al., 2022)
	Mapping vineyard leaf area	(Johnson et al., 2003)
	Automatic detection of vine rows	(Comba et al., 2015)
	Detection of vineyard spatial variation	(Dorin et al., 2024)
	Detection of vineyard spatial variation	(Gatti et al., 2020)
	Detection of vineyard spatial variation	(Di Gennaro et al., 2019)
	Assessment of phenological growth stage of vine plants	(Kasimati et al., 2023)
	Assessment of intra-vineyard vegetation spatial variability	(Matese et al., 2015)

The high-throughput plant phenotyping can also rely on digital applications that represent a new source of quantitative trait data in ecological field studies that offers complementary, multi-dimensional insights into plant communities (Klein et al., 2017; Zieschank and Junker, 2023). In this direction, an automated plant phenotyping system was adapted for mobile use in the field to facilitate ‘digital whole-community phenotyping’ (DWCP), enabling the acquisition of 3-dimensional structures and multispectral data of plant communities. The effectiveness of DWCP was demonstrated through the recording of plant community responses to experimental land-use treatments over a two-year period. In particular, changes in morphological and physiological community properties in response to different treatments were successfully captured (Zieschank and Junker, 2023). Alongside these developments, recent research has demonstrated the potential of hyperspectral and RGB imaging, coupled with machine learning, to further enhance the precision of non-invasive phenotyping of grapevine. We summarize below the main results of some relevant studies on the topic.

Gutiérrez et al. (2018) proposed a novel approach for classifying a large number of grapevine varieties under field conditions by utilizing on-the-go hyperspectral imaging combined with machine learning algorithms. Their experiments, conducted on 30 different varieties, demonstrated that the system effectively classified leaves and could be considered a new non-destructive tool for plant phenotyping in field settings. Similarly, Briglia et al. (2019) investigated whether morphophysiological traits—such as leaf area and leaf water potential—of drought-stressed grapevines could be determined through non-invasive RGB and NIR image-based analysis techniques. Their study successfully modeled plant canopy area estimation based on the pixel count of RGB images from vines subjected to varying levels of drought. Moreover, the results indicated that the NIR and Dark Green color fractions decreased with increasing drought severity, while the Yellow fraction increased.

Montanaro et al. (2024) applied RGB-based phenotyping to detect salt stress responses in potted grapevines, analyzing both the pixel fraction of specific color bands (Yellow, Green, Brown, and Dark Green) and the mean pixel values for R, G, and B. Their findings revealed that a decrease in the relative pixel fraction of Dark Green was associated with an increase in soil electrical conductivity. Additionally, the mean pixel value of R proved to be a reliable predictor of electrical conductivity. Bendel et al. (2020) employed hyperspectral imaging (400–2500 nm) alongside disease detection models to identify grapevines infected with phytoplasma. While the system accurately differentiated between symptomatic and healthy plants (up to 96% accuracy), detecting infected but asymptomatic vines proved more challenging, warranting further investigation.

In a further study on pest detection, del-Campo-Sanchez et al. (2019) demonstrated that combining geometric and computational vision techniques with geomatic products derived from conventional RGB images—acquired by UAV-mounted cameras—enabled more precise segmentation of vegetation affected by the pest *Jacobiasca lybica* healthy vegetation and the ground.

## Smart phenotyping in resilient vine varieties exposed to Cu toxicity

**Figure 1:**
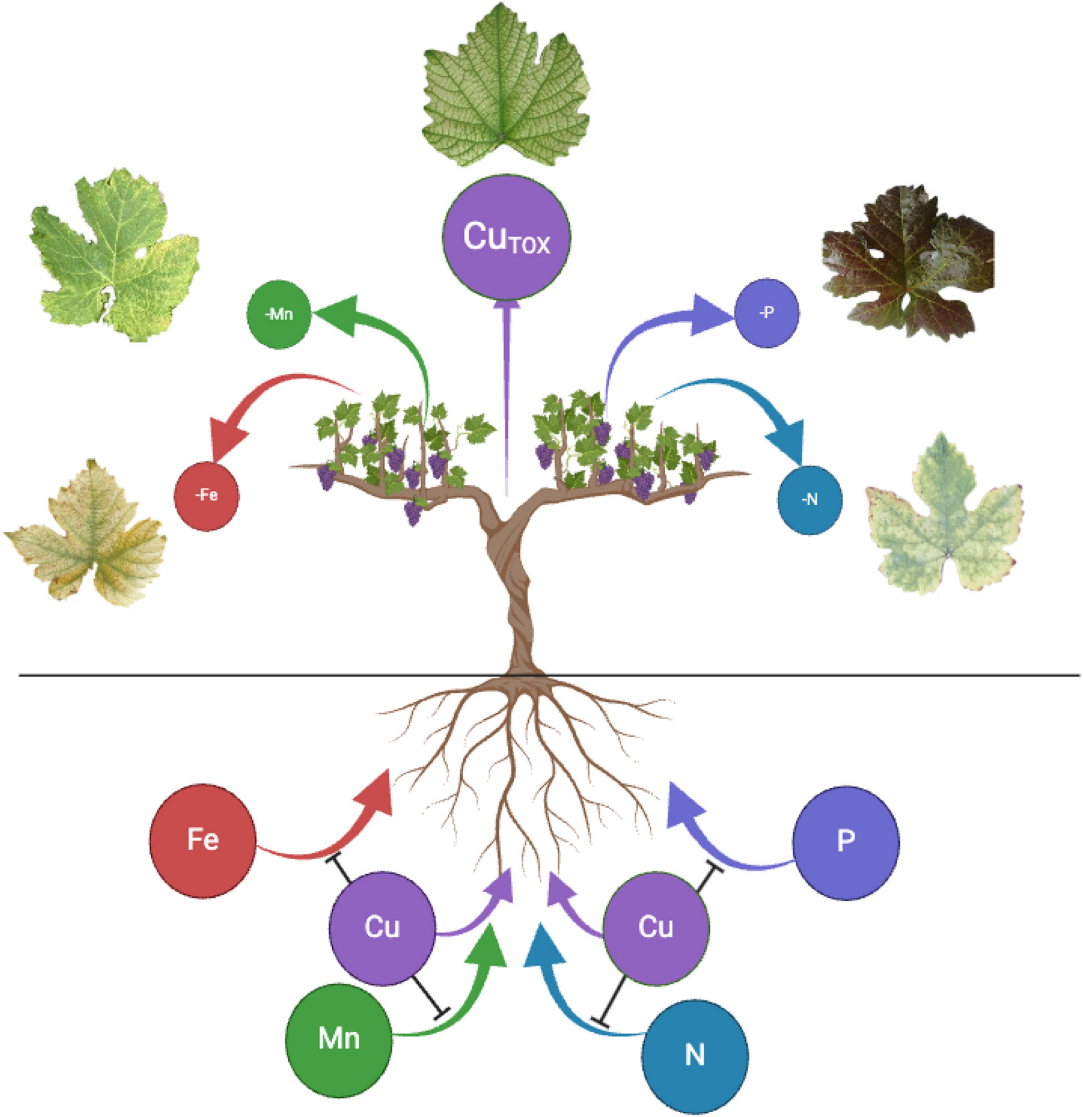
Schematic representation of multiple interactions between Cu and macro- (N and P) and micronutrients (Fe and Mn) at rhizosphere level. High levels of bioavailable Cu in the rhizosphere can result in typical leaf Cu-toxicity symptoms (e.g., chlorosis, wilting and necrosis). In addition, high Cu concentrations can interfere with the acquisition of specific macro- and micronutrients. Copper excess can generate N shortage in plants since it prevents the acquisition of nitrate at root level, by impairing the generation of the proton gradient required for the uptake. The induced-N deficiency is shown by the yellowing of leaf blades. The antagonism between Cu and P is ascribable to a direct interaction of Cu with P acquisition mechanisms, which result indeed inhibited. The induced-P deficiency is shown by bluish darkening of leaf blades. Copper toxicity also induces a fine tuning of the acquisition (e.g., direct competition for the same transporters) and allocation mechanisms of other divalent cations (e.g., Fe and Mn), often creating deficiency conditions that are mainly shown by chlorosis of leaf blades.

The smart phenotyping approach, renowned for its precise, high-throughput, and non-destructive assessments of plant traits, has undeniably provided significant advantages in selecting crop varieties with desirable characteristics like drought and heat tolerance, early maturity, and resistance to pests and diseases (Costa et al., 2019; Langstroff et al., 2022; Maier et al., 2022). This approach might also represent a valuable tool for the identification of i) vine varieties that better adapt themselves to conditions of altered soil fertility, such as those affected by Cu excess, and/or ii) the pathogen-resilient ones (including those already available on the market such as PIWI grape varieties) that best express their resistance traits under varying soil fertility conditions, including Cu toxicity. However, while smart phenotyping is effective in identifying plants manifesting typical Cu toxicity symptoms when grown under high soil Cu availability (*acute toxicity*), its application becomes more complex in cases of lower but supra-optimal and still toxic Cu concentrations, which are very common in vineyard soils, as previously demonstrated by our group (Cesco et al., 2021; Signorini et al., 2021, 2023; Genova et al., 2022). In such instances, high concentrations of Cu in soils can induce both toxicity phenomena and interfere, for example, with the mechanisms underpinning the acquisition of other nutrients (*e.g.*, N, P, Mn) at root level, thus inducing shortages, as described before. In this latter scenario, there is a high risk that visual symptoms are either a mixture of those induced by individual stress (the real – Cu toxicity, and the secondary induced one – nutrient shortage) or rather than exclusively ascribable to the secondary one, thus, making ambiguous the relationship between the primary stressor and the manifested phenotype (**Figure 1**). An example is the bluish darkening of leaf blades, a typical symptom of P deficiency, observed in vine plants exposed to Cu toxicity (Marastoni et al., 2019a). Moreover, a common phenotypic manifestation such as canopy yellowing in plants under stress could be attributed to a multitude of possible causes (*e.g.*, Cu toxicity as well as deficiency of a nutrient). For example, the limited soil availability of N can induce vine plants to manifest a range of symptoms, including low vigor, low berry set, reduced photosynthetic capacity, and generalized yellowing of all leaves and green tissue (Cocco et al., 2021; Verdenal et al., 2021). Furthermore, yellowing of the interveinal area of older leaves is also a symptom exhibited in Mn shortage (Alejandro et al., 2020), often mistaken for Zn or Fe deficiency (Val et al., 1993; Barman et al., 2018). It is worth noting that NDVI values have been used to evaluate canopy health based on the availability levels of certain nutrients, being such applications documented in various crops (*e.g.*, N (Akeem et al., 2018), Fe (Beyyavas et al., 2023). Furthermore, typical symptoms of Cu toxicity such as leaf chlorosis, necrosis and stunted plant development (Cesco et al., 2022; Vasilachi et al., 2023) can also be induced by heavy metals such as Cd and Pb, which are commonly found in cultivated soils (Rashid et al., 2023), adding further complexity to the system. Given these factors, it appears that automated canopy assessments can effectively highlight plant distress in such circumstances, though they may not definitively identify the underlying cause. In this context, it is important to recognize that the agricultural environment is a complex system where multiple factors often simultaneously limit the full productive potential of a cultivated plant species, as seen in cases of concurrent sulfur (S) and iron (Fe) deficiencies (Astolfi et al., 2021). Moreover, low availability of one nutrient can impair the acquisition mechanisms of others (*e.g.*, Fe for nitrate (Agnolon et al., 2002), N for Fe (Nikolic et al., 2007), S for Fe (Astolfi et al., 2021)), further complicating the sequence of events at the plant’s adaptive response to Cu toxicity and the characteristics of the resulting phenological manifestation.

Although the application of smart phenotyping in characterizing resilience to Cu toxicity in grapevine plants is complex and challenging due to the intricate network of stress responses involved, it is important to note that trained professionals can unequivocally identify the primary stressor when provided with specific details (*e.g.*, canopy location of affected leaves, type and intensity of symptoms on nearby indicator plants, including weeds, symptoms at the root level, analytical data from plant tissues, etc.). This aspect opens possibilities for effectively utilizing data obtained through smart technologies when integrated (*data fusion*) with other methods (such as analytical data collected also with destructive approaches) and transformed in actionable information (Mazzetto et al., 2010). However, the entire procedure becomes enormously more complicated as the number of data sources increases, along with the relative levels of uncertainty (*blur*) associated with each source. A clear example is found in diagnostic processes that rely on data derived from image analysis. In situations like these, where purely deductive approaches are practically infeasible, artificial intelligence, particularly exploiting neural networks, can provide significant cognitive support. Nevertheless, before they can be used to recognize patterns and complex relationships in data, neural networks must be trained through a training algorithm with a very high number of input/output correspondences known *a priori*. Through this learning step, the neural network may be able to autonomously identify patterns and relationships in the data, extracting general rules or abstract representations. Examples include the automatic identification of the vegetative stage and phytosanitary conditions of crops, as well as preventive diagnostics of various physio-pathologies and pest infections (Kamilaris and Prenafeta-Boldú, 2018; Popescu et al., 2023), which show promising elements for potential use in the context of vine plants and the soil Cu issue. However, it is noteworthy that these inferential procedures, based on inductive logic, do not guarantee absolute certainty in the validity of results, even when they are considered highly probable. The reliability of these outcomes depends on the quality of the prior training process, which is influenced by the number of input/output combinations and the completeness and reliability of the information used (Mazzetto et al., 2010). For these reasons, the contribution of the neural inferences of artificial intelligence to smart phenotyping in the viticultural sector (*i.e.*, for the breeding of new rootstocks and varieties) should still be considered as a decision support system in which the final decision remains with an operator. Although proposed for breeding programs, it cannot be excluded that in the near future, advancements in these technologies could be applied in routine agricultural practices, such as for smart fertilization and/or the smart management of phytosanitary defense in vineyards. Moreover, a step forward towards levels of the automation process that implements creativity and design features can probably be made with the more widespread application of generative artificial intelligence through particularly advanced neural processes with machine learning capable of generating original data or content. It should be added that the applications of this AI technology might offer significant advantages in analyzing complex cases in viticulture, through the simultaneous analysis of large numbers of individuals, leveraging big data, and providing rapid response times.

## Conclusions

The challenges linked to climate change and the need for increasingly sustainable viticulture underscore the essential need for vine varieties/cultivars that are more resilient to various abiotic and biotic stresses, which are gradually becoming more severe. In this selection process, the frequent alteration of vineyard soil fertility due to excessive Cu availability necessitates a careful evaluation, especially in viticulture-suited areas. This includes not only assessing the traits related to Cu toxicity resistance in both old and new rootstocks and sprouts but also examining the expression levels of pathogen-resilience traits in new vine varieties or cultivars when exposed to these altered soil conditions. Moreover, the complexity of Cu-toxicity symptoms in plants makes this process particularly challenging, especially for non-acute toxicity conditions, which represent the majority of cases. In this context, the applications of smart technologies associated with artificial intelligence neural networks can make a notable contribution both in terms of the number of individuals analyzed and response time. However, despite their undeniable advantages, the inherent uncertainties in the inductive logic inferential procedures of neural networks mean that artificial intelligence should still be viewed as a decision support system, rather than a replacement for the operator’s final judgment.

Indeed, by improving phenotyping techniques, researchers can more accurately assess how different plant varieties respond to environmental stresses, nutrient availability, and other critical factors. This knowledge is crucial for developing more resilient crops, optimizing agricultural practices in the face of climate change. Emphasizing the importance of such studies will foster innovation and support the development of sustainable agricultural systems. Moreover, these technologies are highly applicable to both viticulture and sustainable agriculture, offering enhanced precision in disease detection, optimized resource management, and data-driven decision-making, ultimately improving sustainability and productivity.

## Data Availability

The original contributions presented in the study are included in the article/supplementary material. Further inquiries can be directed to the corresponding author.
